# Experiences and results from using a novel clinical feedback system in routine stoma care nurse follow-up of patients with an ostomy: a longitudinal study

**DOI:** 10.1186/s41687-023-00573-z

**Published:** 2023-03-13

**Authors:** Kirsten Lerum Indrebø, Anny Aasprang, Torill Elin Olsen, John Roger Andersen

**Affiliations:** 1 Department of Surgery, Førde Central Hospital, Svanehaugvegen 2, 6812 Førde, Norway; 2 Centre of Health Research, Førde Hospital Trust, Førde, Norway; 3 Western Norway University of Applied Sciences, Førde, Norway; 4 Western Norway University of Applied Sciences, Bergen, Norway; 5 Department of Surgery, Haukeland University Hospital, Bergen, Norway

**Keywords:** Ostomy, Patient-reported outcomes, Clinical feedback system, Stoma care nurse, Outpatient follow-up, Ostomy adjustment, Health-related quality of life

## Abstract

**Background:**

A faecal or urinary ostomy may be lifesaving. However, it involves significant bodily change, and the adjustment process to life with an ostomy includes a broad spectre of physical and psychosocial challenges. Thus, new interventions are needed to improve adaptation to living with an ostomy. This study aimed to examine experiences and outcomes using a new clinical feedback system with patient-reported outcome measures in ostomy care.

**Methods:**

In this longitudinal explorative study, 69 ostomy patients were followed by a stoma care nurse in an outpatient clinic, using a clinical feedback system postoperatively at 3, 6 and 12 months. The patients responded electronically to the questionnaires before each consultation. The Generic Short Patient Experiences Questionnaire was used to measure patient experiences and satisfaction with follow-up. The Ostomy Adjustment Scale (OAS) measured adjustment to life with an ostomy, and the Short Form-36 (SF-36) assessed the patient's health-related quality of life. Longitudinal regression models with time as an explanatory (categorical) variable were used to analyse changes. The STROBE guideline was applied.

**Results:**

The patients were satisfied with their follow-up (96%). Especially, they felt they received sufficient and individualised information, were involved in treatment decisions, and benefited from the consultations. The OAS subscale scores for 'daily activities', 'knowledge and skills' and 'health' improved over time (all *p* < 0.05), as did the physical and mental component summary scores of the SF-36 (all *p* < 0.05). Effect sizes of changes were small (0.20–0.40). Sexuality was the most challenging factor reported.

**Conclusions:**

The clinical feedback system could be helpful because outpatient follow-ups for ostomy patients may be more tailored when clinicians use clinical feedback systems. However, further development and testing are needed.

## Abstract (plainenglishlanguage)

Studies show that patients struggle with adjustment to the ostomy in a broad spectre of physical and psychosocial factors, and stoma care nurse follow-up is necessary. Mapping each patient`s most important challenges during a short outpatient follow-up consultation may be difficult. Thus, missing information on individual factors affecting adjustment and quality of life can result in underreported problems and unclear communication between patients and stoma care nurse. The study aimed to explore patient satisfaction and experiences using digital questionnaires before each consultation in stoma care nurse ostomy follow-up and to study adjustment to ostomy and health-related quality of life in routine follow-up 3,6 and 12 months after ostomy surgery. The study results showed that the patients were satisfied with their follow-up using questionnaires before consultations (96%). Especially, they felt they received sufficient and individualised information, were involved in treatment decisions, and benefited from the consultations. Especially the life areas “daily activities”, having “knowledge and skills”, “health”, and the patient`s health-related quality of life improved during the first year after the operation. Thus, patient reported outcomes/clinical feedbacksystem is a promising method for follow-up of ostomy patients because it may promote better discussions during the consultation and tailor the patient’s adjustment trajectory more precisely than without such a system.

## Background

Ostomy surgery is necessary for about 1900 people annually in Norway, owing to colorectal cancer, inflammatory bowel disease (IBD), infections, incontinence and several other diagnoses [1]. With an ostomy, the urine or faeces enter an external pouch on the abdomen, and patients must adjust to bodily changes after the operation [2]. These changes in appearance and bodily function can influence physical, psychological and social life [3–6], as well as health-related quality of life (HRQoL) [7, 8]. 

Sufficient knowledge and the skills to carry out ostomy care and psychological support are essential to adjusting to life with an ostomy and enjoying HRQoL. A study by Notter et al. suggests the importance of a high degree of individualised follow-up following ostomy [9]. The physical and psychosocial adjustment to body changes after an ostomy operation is an individual process that lasts for years. Thus, the patient needs a long time individualised follow-up. Several studies have shown that stoma care nurses (SCNs) are central in the education and long-term follow-up of stoma patients and that patients and SCNs need to communicate effectively according to the patient's needs. [3, 4, 9–14]. To promote the patient's adjustment to life with an ostomy, the SCN needs knowledge of the patient's experiences with having an ostomy in their everyday life.

Consequently, it would be helpful to allow each patient to prepare for follow-up consultations and bring their experiences, knowledge and challenges into their communication with the SCN. However, patients do not always know what to ask about, and the SCN may not always grasp their patients' struggles [9]. Unclear communication may result in problems that are underreported at consultations.

Several interventions promote better adjustment to ostomy and better QoL following ostomy surgery. For example, education programs, telephone or text message follow-up [15–17], face-to-face education sessions [18] and participating in ostomy self-care programs [19] have all been found useful. Another finding is that the communication between patients and SCN is a significant factor in the adjustment process [3]. Still, there is a gap in the literature on using patient-reported outcomes (PROs) in routine clinical consultations with ostomy patients. There is also a lack of longitudinal studies studying the adjustment process in patients who regularly follow up with SCNs.

**Fig. 1:**
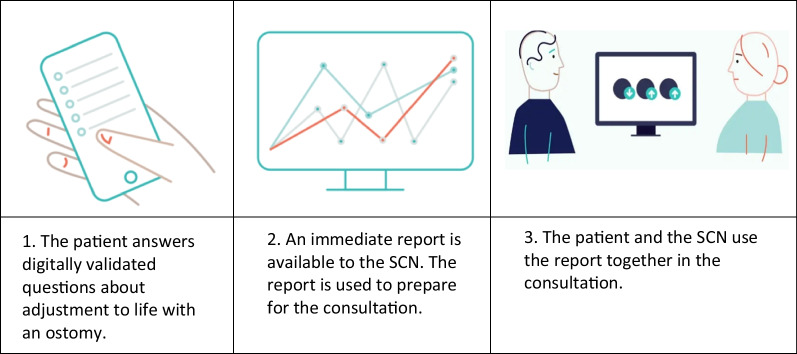
Ostomy adjustment system

A promising tool for preparing and conducting these consultations is utilizing electronic PROs to monitor the patient's treatment progress over time [20]. PROs can be easily implemented in a clinical feedback system (CFS) in patient consultations, using an electronic device displaying results with user-friendly graphs [21] (Fig. 1). The CFS can be used as a communicational tool to improve user involvement in treatment decisions and measure the patient's progress in treatment over time [20, 22–25].

The current study aimed to explore experiences and results from a novel CFS in ostomy patients receiving SCN follow-up in a routine clinical setting. We report experiences and satisfaction with patient care using the CFS, patient trajectories of change in adjustment to life with an ostomy, and comparisons of generic HRQoL between patients with an ostomy and norm scores from a general population. We also report the experiences and reflections of the SCNs on the development and use of the CFS.

## Methods

In this longitudinal study, we included patients who had undergone urostomy, colostomy, or ileostomy operations attending the regular follow-up programme of the outpatient ostomy clinic at the Department of Surgery from September 2017 to December 2021. The inclusion criteria were age > 18 years; to have had a colostomy, ileostomy or urostomy for ≥ 3 months; and to be able to speak, read and write Norwegian. The SCNs considered whether the patients filled the inclusion criteria. Those who fulfilled the criteria received a written information letter about the study on three weeks of postoperative outpatient consultation. A written consent form was added to the information. The study included the participants for four years, and each patient was followed for 12 months postoperative". The study followed the STROBE guideline.

Our power calculation was based on a two-sided paired test (effect size = 0.4, a correlation between measures of 0.3, 90% power, p ≤ 0.05), the results of which indicated that at least 68 paired observations would be required to detect reasonably robust 95% confidence interval (CI) estimates of changes on the primary outcome of interest: Ostomy Adjustment Scale (OAS) [26]. No minimally important effect sizes have been defined for the OAS; thus, we relied on research and consensus for PRO measures in general [27].

### Clinical feedback system

Planning and implementing the new intervention for outpatient follow-up of ostomy patients using the CFS involved several components, including the selection of instruments, development of the digital version, user involvement, planning and implementation of the follow-up consultations, and the documentation of results in the patient's journal. A more detailed overview is available in the study protocol [28].

Three SCNs followed up the patients at the 3-, 6- and 12-month postoperative intervals in an outpatient clinic, using electronic PROs and a CFS [28]. The PROs had to reflect the patient's adjustment process, HRQOL, and important patient experiences and satisfaction with the outpatient follow-up consultations, and the scales must have been validated in Norway. The follow-up was conducted according to national recommendations for the follow-up of ostomy patients in Norway [29]. Participants completed an electronic sociodemographic and clinical form, the OAS and the Short Form-36 (SF-36), prior to or occasionally during their postoperative 3-, 6-, and 12-month consultations with the SCN. The patient and SCN discussed the answers during their consultation, worked together on new interventions and planned further follow-up. After each consultation, the patients responded to a paper version of the Generic Short Patient Experiences Questionnaire scale (GS-PEQ), and the SCN responded to an electronic form. Using a paper version could mean less patient burden because the patient did not need to log in with Bank Id again after the consultation to answer a digital questionnaire. The OAS was previously cross-culturally adapted in Norway according to guidelines for cross-cultural adaptation of self-report measures [30, 31]. The GS-PEQ was developed in Norway [32]. The CFS and its implementation plan for clinical practice have been described in detail elsewhere [28] (Fig. 1).

#### Documentation of results in the patient's electronic journal

As ostomy follow-up was part of a research project, the questionnaires were not incorporated into the patient's electronic hospital journal. The patients' answers were reported as bars (SF-36), graphs (OAS) and reports (clinical forms) on their screens. The PROs and results from the clinical component of the consultation were documented in the patient's electronic journal, together with the interventions that the patient and SCN agreed on. The patient can read the SCN's report in the electronic journal.

#### User involvement during the development process

The questionnaire package was discussed with the patient user panel and approved by them, focusing on the burden of answering 96 items and the experience of responding to the questionnaires at home. During the study, the SCNs discussed the development of the OAS subscales and patient expectations from a consultation, including using the CFS. Feedback indicated that using the questionnaires made it easier to discuss self-esteem/body image and psychological/existential factors, enabling them to be viewed as 'whole persons'.

#### Electronic platform and security

Our in-house expertise on digital platforms and statistical programmes helped us communicate as precisely as possible in "technology language" with the private company developing the electronic version. In Norway, bankID is a system for the identification and storage of sensitive personal data. To access this data in the hospital, using a code device or a cell phone and having access to a mobile network is necessary. We account for the fact that some patients forgot to bring their bank ID code device with them. In addition, mobile signal strength varied in the region around the hospital where the research was done. Due to these limitations, access to bank ID information was not always feasible. Thus, we created a reserve solution giving the patient one-time login codes for each questionnaire.

### Variables

#### Sociodemographic and clinical forms

The sociodemographic and clinical forms were based on theory [29, 33] and the long-term experiences of SCNs in the follow-up of ostomy patients. The same forms were used in the Norwegian validation study of the OAS [31].

The form completed by the patients included items on age (continuous variable), gender (male or female), marital status (married/cohabiting or living alone, and education (low [< 13 years] or high [≥ 13 years]).

The form completed by the SCN included items on time since surgery (< 1 year or > 1 year), diagnosis (ulcerative colitis/Crohn's disease, cancer or other diseases) and ostomy type (colostomy, ileostomy, urostomy or two ostomies).

#### Patient experiences scale

At the start of the study, we used a nonvalidated questionnaire about patient experiences and satisfaction with follow-up. During the study, we discovered a validated Norwegian scale, which reflected the patient's experiences and satisfaction with outpatient consultations very well. The two scales mainly contained the same areas, but due to recommendations to use validated scales, we decided to change the scale during the study, and the responses on the nonvalidated scale were not analysed.

#### Generic short patient experiences questionnaire

The GS-PEQ was used to measure patient experiences. The scale contains questions about patient satisfaction and experiences with somatic outpatient services in Norway [34]. It includes 10 generic core items about dimensions of the patient's experiences in using specialist health care services. The areas covered by the scale are outcome (two items), clinician services (two items), user involvement (two items), incorrect treatment (one item) and information (one item). The answers are scored on a five-point response scale from 1 = 'Not at all', 2 = 'To a small extent', 3 = 'To a moderate extent', 4 = 'To a large extent', and 5 = 'To a very large extent'. In addition, 'Not applicable' was a response option. The 10 items in the GS-PEQ have been rated highly important and relevant in research [35]. The GS-PEQ items about what happened in the consultation were essential in evaluating SCN follow-up. The GS-PEQ was developed in Norway [32].

#### Ostomy adjustment scale

The OAS is a 34-item multidimensional scale that measures a patient's subjective adaptation to physical, psychological and social changes after ostomy surgery. The OAS comprises seven subscales measuring adaptation to ostomy relating to daily activities, knowledge and skills, self-esteem/body image, psychological/existential aspects, health, health professionals and sexuality [36]. Notably, it includes items about employment status, leisure, trust in ostomy equipment, and general description of life with an ostomy. The scale also records patients' opinions on the instructions they received about their ostomy, their self-image and social functioning, their feelings about the ostomy, their relationship with health professionals and their sexuality in relation to it [37]. The OAS is scored on a Likert scale from 1 (strongly agree) to 6 (strongly disagree). We used a total mean score and subscores ranging from 1 to 6. A pragmatic thumb of rule based on clinical experience is that subscores higher than 4.35 indicated good adjustment, scores from 2.67 to 4.34 showed some challenges and scores from 1 to 2.66 indicated low adjustment[36]. Previous reports on the reliability and validity of the OAS demonstrated acceptable internal consistency and test–retest reliability [37–39]. Previous studies also support the instrument's construct validity [37–39]. Mary Ellen Olbrisch, the researcher who developed the instrument, permitted us to freely use the OAS. "The OAS was cross-culturally adapted in Norway according to guidelines for the cross-cultural adaptation of self-report measures" [31, 30].

In the current study, the participants responded electronically to single items before the consultation. We divided the OAS scale into clinically meaningful subscales during the study period and analysed our research results according to the subscales. To divide the OAS into subscales, SCNs and researchers first divided the scale into clinically meaningful subscales. After that, the model was statistically tested using confirmatory factor analysis [36].

#### Short form-36

The SF-36 is a well-validated, generic health scale that measures outcomes (health phenomena) known to be the most directly affected by disease and treatment [40]. The SF-36 has eight subscales measuring physical functioning, physical role limitations, emotional role limitations, bodily pain, general health, vitality, social functioning, emotional role functioning and mental health. The instrument has two summary scores: a physical component score (PCS) reflecting the domains of physical function, physical role function, pain, and general health, and a mental component score (MCS) reflecting the domains of vitality, social function, emotional role functioning and mental health. The SF-36 scores are presented from 0 to 100, with higher scores reflecting better HRQoL. The Norwegian version of the SF-36 has satisfactory reliability and validity [41], and Norwegian population norm scores for the SF-36 stratified by age and gender were derived from a recent publication [42].

#### The nurse's experiences and reflections on the PRO/CFS

Experiences of the time spent in each consultation were gathered from the SCN's appointment list in the hospital's administrative system.The SCN's experiences and reflections on using the CFS were discussed in meetings between the SCNs and the developers and summarized in reports. If necessary, minor adjustments in the intervention were made continuously. Some of the thoughts and lessons are presented further.

### Data analyses

The characteristics of the sample (n = 69) were presented as numbers and percentages, except for age which was presented as mean and standard deviation (SD). Data missing from the questionnaires was handled according to the procedures described for each questionnaire [37, 43]. The OAS and the SF-36 scores at 3, 6 and 12 months after the operation were presented as means with 95% CIs. To study changes in the OAS and the SF-36 scores, longitudinal mixed-effect regression models with time as an explanatory variable were used, with exact two-sided p-values. A one-sample t-test was used to study differences in SF-36 scores in the patient group versus the general population. Effect sizes for change in OAS and SF-36 were calculated by subtracting the average scores between time points divided by the SD by the 3-month consultation. Effect sizes for differences in the SF-36 scores between the patients and the general population were calculated by subtracting the patients' average scores from the average population scores and dividing them by the SDs from the study population. All effect sizes were judged against the standard criteria proposed by Cohen [45] as follows: trivial (< 0.2), small (0.2 to < 0.49), moderate (0.5 to < 0.79), and large (≥ 0.8) [44]. In the analysis of patient experiences and satisfaction with care received, descriptive results (number and percentage) for each item of the GS-PEQ at 1-year follow-up were presented. SPSS software (version 25; IBM, Armonk, NY) was used for all analyses.

## Results

The sociodemographic and clinical data are presented in Table 1. Of the patients, 35 (51%) responded to the questionnaires electronically from home, 17 (24.6%) answered at the hospital just before the consultation and 17 (24.6%) answered the questionnaires during the consultation. None used a paper version. The patients used approximately 20 min to answer the questionnaires. Each consultation lasted 1 h unless patients needed help answering, in which case the consultation was up to 1.5 h. All invited patients agreed to participate in the study (Tables 2 and 3), but it was not complete data on all measure points. Reasons for not answering at 3 or 6 months were technical problems, changes of appointments, and restrictions owing to the Covid-19 pandemic. The response rates at twelve Month measure were 100% in the subscales "daily activities", "knowledge and skills", "self-esteem/body image", "psychosocial/existential and 97% on health, 88% on "health professionals and 64% on "sexuality".

**Table 1:** Demographic and clinical characteristics (n = 69)

Variable	Value
Age, mean years (range)	62.71 (20–86)
*Gender, n (%)*	
Women	25 (36.2)
Men	44 (63.8)
*Marital status, n (%)*	
Married/cohabitant	46 (66.7)
Living alone	23 (33.3)
*Type ostomy, n (%)*	
Ileostomy	21 (30.4)
Colostomy	34 (49.3)
Urostomy	8 (11.6)
Two ostomies (colo and uro)	6 (8.7)
*Diagnosis n (%)*	
Cancer	41 (59.4)
Inflammatory bowel disease	15 (21.7)
Other diseases or conditions	13 (18.8)
*Education, n (%)*	
Primary school/senior high school/college	52 (75.4)
University college/university	16 (23.2)
Missing	1 (1.4)

**Table 2:** Short patient experiences questionnaire at 12 months follow-up: crude numbers (n = 48)

Items	Not at all	To a small extent	To a moderate extent	To a large extent	To a very large extent	Not applicable
Did the clinicians talk to you in a way that was easy to understand?	0	0	0	8	40	0
Do you have confidence in the clinicians’ professional competence?	0	0	0	4	44	0
Did you get sufficient information about your diagnosis/your afflictions?	0	0	0	11	35	2
Did you perceive the treatment you received as suited to your situation?	0	0	2	10	36	0
Were you involved in any decisions regarding your treatment?	0	1	3	9	34	1
Did you perceive the institution’s work as well organised?	0	0	1	10	31	6
Do you believe that you were in any way given the wrong treatment (according to your own judgement)?	37	3	0	0	5	3
Overall, were the help and treatment you received at the institution satisfactory?	0	0	1	12	34	1
	Not at all	Yes, but not so long	Yes, quite long	Yes, much too long	–	Not applicable
Did you have to wait before you were admitted for services at the institution?	39	4	0	4	0	0
	No benefit	Small benefit	Some benefit	Great benefit	Huge benefit	Not applicable
Overall, what benefit have you had from the care at the institution?	0	0	3	18	27	0

**Table 3:** Ostomy adjustment scores over time

Scores	3 months, mean (95% CI)	6 months, mean (95% CI)	12 months, mean (95% CI)	Effect size*	*p*-Value **
Sum score total	4.44 (4.27, 4.67)	4.62 (4.43, 4.81)	4.72 (4.53, 4.90)	0.33	0.008
Daily activities	4.06 (3.79, 4.30)	4.30 (4.06, 4.54)	4.42 (4.18, 4.65)	0.36	0.008
Knowledge and skills	5.14 (4.87, 5.36)	5.21 (4.98, 5.44)	5.47 (5.26, 5.68)	0.37	0.025
Self-esteem/body image	4.72 (4.51, 5.03)	4.90 (4.66, 5.15)	4.98 (4.74, 5.22)	0.23	0.165
Psychological/existential	4.07 (3.82, 4.39)	4.36 (4.09, 4.63)	4.40 (4.05, 4.57)	0.27	0.138
Health	4.97 (4.73, 5.29)	4.92 (4.65, 5.18)	5.32 (5.05, 5.55)	0.34	0.016
Health professionals	5.40 (5.12, 6.67)	5.41 (5.15, 5.67)	5.34 (5.10, 5.59)	0.06	0.889
Sexuality	2.88 (2.44, 3.41)	3.20 (2.76, 3.66)	3.21 (2.76, 3.65)	0.20	0.481

*CI* Confidence interval

Number of observations: 3 months, n = 48; 6 months, n = 59; 12 months, n = 69

*Effect sizes are based on the differences between the 3-month versus the 12-month scores divided by the standard deviation of the 3-month scores. Effect sizes < 0.2 are considered trivial, from 0.2 to < 0.5 are considered small, from 0.5 to < 0.8 as moderate and ≥ 0.8 as large

**p-Values are for overall changes over time

### Patient experiences and satisfaction with PRO/CFS

Of the participants, 48 answered the GS-PEQ questionnaire, and the first 29 responded to a non-validated form about satisfaction with care. First, almost all the patients indicated that the SCN talked to them in a way that was easy to understand. Second, they received sufficient information about their diagnosis and condition. Third, all participants had confidence in the clinicians' professional competence. Fourth, the treatment was suited to their situation, and they were involved in any treatment decisions; and fifth, they reported a 'great' or 'huge' benefit from the care they received (Table 2).

### Trajectories of change in HRQoL and adjustment to life with an ostomy

#### Adjustment to life with an ostomy

The participants showed significant improvement in OAS total sum score from 3- to 12 months postoperatively (*p* = 0.008), with an effect size for change of 0.30. The following subscale scores improved significantly from 3- to 12 months postoperatively: daily activities (*p* = 0.008), knowledge and skills (*p* = 0.025) and health (*p* = 0.016). The effect sizes of change were small for the OAS sum score and the subscales scores for daily activities, knowledge and skills, health, self-esteem/body image and psychological/existential, and were trivial for health professionals and sexuality. Scores for the sexuality subscale indicated challenges throughout the first year post-ostomy, and scores were not significantly better at 12 months. Thus, sexuality was the greatest patient-reported challenge (Table 3).

#### Health-related quality of life

MCS and PCS showed significant positive change 12 months postoperatively compared with the 3- and 6-month scores, with small effect sizes. Results from the subscales of physical functioning, physical role functioning and emotional role functioning were significantly better at 12 months than at 3 and 6 months, but the effect sizes were small. In all other SF-36 subscales, the effect sizes of the changes were trivial (Table 4). Compared to norms for the Norwegian population, the PCS and MCS scores were lower at 12 months postoperatively, but the effect sizes were small. The effect sizes for the subscales were also small (physical functioning, physical role functioning and emotional role functioning) or trivial (bodily pain, general health, vitality, social functioning and mental health) (Table 5).

**Table 4:** Patients Short Form-36 scores over time

Scores	3 months, mean (95% CI)	6 months, mean (95% CI)	12 months, mean (95% CI)	Effect size*	*p*-Value**
Physical component score	61.16 (55.75, 66.56)	68.75 (63.71, 73.79)	68.86 (62.06, 71.65)	0.41	0.015
Physical function	68.76 (62.26, 75.26)	77.28 (71.18, 83.39)	75.04 (69.17, 80.91)	0.31	0.011
Physical role function	35.01 (24.07, 45.95)	50.94 (40.94, 60.95)	51.45 (42.07, 60.83)	0.43	< 0.001
Pain	73.07 (66.20, 79.94)	80.86 (74.51, 87.21)	74.67 (68.67, 80.68)	0.06	0.054
General health	68.18 (62.20, 74.15)	66.28 (60.69, 71.87)	66.40 (61.07, 71.72)	− 0.08	0.752
Mental component score	69.32 (64.17, 74.47)	76.84 (72.08, 81.58)	73.35 (68.88, 77.82)	0.21	0.025
Vitality	53.14 (46.89, 59.39)	58.18 (52.33, 64.03)	56.33 (50.79, 61.86)	0.14	0.260
Social function	76.88 (70.63, 83.13)	82.88 (77.16, 88.60)	80.97 (75.61, 86.34)	0.16	0.217
Emotional role function	66.01 (55.60, 76.42)	83.84 (74.34, 93.33)	74.40 (65.64, 83.15)	0.20	0.016
Mental health	81.29 (77.30, 85.27)	82.78 (79.08, 86.47)	81.68 (78.21, 85.15)	0.03	0.747

*CI* Confidence interval

Number of observations: 3 months, n = 46; 6 months, n = 58; 12 months, n = 69

*Effect sizes are based on the differences between the 3-month versus the 12-month scores divided by the standard deviation of the 3-month scores. Effect sizes < 0.2 are considered trivial, from 0.2 to < 0.5 are considered small, from 0.5 to < 0.8 as moderate and ≥ 0.8 as large

**p-Values are for overall changes over time

**Table 5:** Patients’ Short Form 36 scores at 12-months follow-up versus norm scores

Scores	12 months, mean (standard deviation)	Norm scores, mean	Effect size	*p*-Value
Physical component score	68.86 (22.18)	74.66	− 0.26	0.033
Physical function	75.04 (26.10)	83.55	− 0.33	0.009
Physical role function	51.45 (41.10)	71.26	− 0.48	< 0.001
Pain	74.67 (26.14)	72.97	− 0.07	0.591
General health	66.40 (22.58)	70.86	0.20	0.106
Mental component score	73.35 (20.13)	79.26	0.29	0.017
Vitality	56.33 (25.82)	61.09	− 0.18	0.130
Social function	80.97 (22.95)	87.28	− 0.17	0.026
Emotional role function	74.40 (37.98)	86.59	− 0.32	0.010
Mental health	81.68 (15.21)	82.06	− 0.03	0.836

Norm scores were adjusted for age and gender to reflect the same distribution as the study sample

Effect sizes were calculated by subtracting the mean score of the population norm from the mean score of the patient group divided by the standard deviation of the patient group. Effect sizes < 0.2 are considered trivial, from 0.2 to < 0.5 are considered small, from 0.5 to < 0.8 as moderate and ≥ 0.8 as large

Number of observations = 69

### SCN'observations during followup

#### Follow-up consultation

The procedure for the follow-up consultations was developed in detail before we started the project [28]. However, after four years of implementing the consultations, it was clear that their development was an ongoing process. The implementation of the consultations differed from patient to patient because they were tailored to each patient's answers to the questionnaires and individually adapted to the patient's preferences for discussing their challenges. Practical issues needed addressing, such as having an appropriate place to answer the questionnaires in the outpatient clinic waiting area. We had to remember to change the questionnaire availability time for patients who changed their appointment.

Altogether, using PRO/CFS in patient consultations made it easier for the patients to bring up and discuss difficult themes, especially self-esteem, existential/psychological challenges, and sexuality. We used the single-item version of OAS in the consultations. Using this version could, in some consultations, result in specific questions dealing with the same topic being discussed several times in the consultation. In the future, using subscales could be a promising method to avoid discussions about the same topic several times.

After the consultation, a paper version of the GS-PEQ may have resulted in a higher response rate because the patients did not have to log in with Bank ID again to answer the questionnaire. Due to login challenges, the consultation could last longer than planned. Therefore, in the future, the login procedure should be more straightforward.

## Discussion

This study reports the initial results of using a new CFS for people with an ostomy. User satisfaction was high, with 96% of the patients reporting being satisfied to a large extent or to a very large extent with the help they received. Patient adjustment to life with an ostomy improved significantly from 3 to 12 months postoperatively on the subscales of daily activities, knowledge and skills, and health. Sexuality was clearly the most challenging life domain with little improvement over time. Overall, HRQoL, as measured with the SF-36 summary scores, improved significantly over time but remained slightly below general population norms 12 months after surgery. To our best knowledge, this is the first study of its kind in ostomy care. Thus, a direct comparison of our results with others is not feasible. Consequently, we compare our results with those from other studies that might be informative**.**

### Patient experiences and satisfaction with using the CFS

The high scores on the GS-PEQ and the OAS subscale for 'health professionals' indicate that the CFS is a promising communication tool in the nurse-led follow-up of ostomy patients. However, scores could also have been high because patients may have been 'eager to please' because their future follow-up may have been with the same SCN. Of the patients, 24.6% responded to the OAS during the consultation, and their answers about their relationships with health professionals may have been less honest than those of patients answering before their consultation. Another factor was that the researcher was one of three SCNs conducting the follow-up. The use of PROs has been reported for other patient groups, such as in a longitudinal study among 100 home dialysis patients who received nurse-led outpatient follow-up every third month, including the reporting of electronically PROs before and after the consultations [45]. The study results indicated positive experiences for patients and nurses using PROs. Patients were satisfied with the nurses' assistance, and the level of satisfaction with care was stable over time. About 40% reported that they felt more supported and had a better understanding of their situation and how they could improve it. In another nurse-led randomised controlled pilot trial among patients with diabetes [54], 32.1% of participants stated that completing PROMs led to discussions of diabetes-related challenges that would not otherwise have occurred. However, a Swedish study [4] of regular 3-, 6- and 12-month postoperative follow-ups of ostomy patients without CFS also showed high OAS mean scores in the three single items about health professionals. Measuring patient experiences is challenging owing to the complexity of the consultation. For example, it may be difficult for the patient to separate their experiences of the instruments and methods used and the SCN's competence and ways of communicating and teaching. A future research option could be a randomised controlled study of patients receiving follow-up with CFS compared with patients subject to standard follow-up. However, our CFS intervention must first be further developed and tested at more ostomy clinics.

### Patients' trajectories during the first postoperative year

In the current study, the patients were enrolled three months after their ostomy surgery. We found that their OAS sum score improved from three months to one year postoperative. Comparing our findings with other studies on improving OAS and SF-36 scores during the first postoperative year is difficult because few longitudinal studies have used PRO/CFS. A case–control study from Denmark [15] studied the effect of an education programme on OAS sum scores from baseline (before hospital discharge) to 3- and 6 months postoperative. The OAS sum score was lower than in the current study at these points, possibly because of differences in the study population and follow-up schedules.

Our study showed small but significant effect sizes reflecting improvements in daily activities, knowledge and skills, and health areas between 3 and 12 months postoperatively. Self-esteem/body image and psychological/existential factors showed small effects, but these were not significant. One explanation could be that the greatest change happens between hospital discharge after surgery and three months postoperatively [15]. However, adjustment to living with bodily changes may be complex and lengthy because the various aspects of this influence each other. For example, the ostomy, the area surrounding it and its function may change owing to changes in behaviour or body shape. These may result from dietary changes, weight gain or loss, more physical activity, travelling, resumption of work and participation in new social settings. The patient continually learns how to prevent complications, such as parastomal hernia, leakage and sore skin, possibly changing their clothing style, and how to deal with unpleasant sounds from their ostomy in social settings. Other studies have shown that even patients living with an ostomy for several years lack the knowledge to manage leakage and sore skin [46]. One focus group among patients with 1 to 3 years of experience with colo- or ileostomy found that patients still did not feel comfortable with their new body [47]. Similar findings were also reported from another focus group study among six young people with ostomies owing to IBD [48]. The participants reported uneasy feelings about the ostomy, such as embarrassment and having to change their wardrobe to conceal the ostomy bag, causing them to feel different from their peers, restricted in activity and clothing choices, and experience loss of control. A study among colostomy patients showed that patients with high levels of knowledge and independence had higher psychosocial adjustment than those with less competence [49]. Of the participants in our study, 59.6% had a cancer diagnosis, and most of the study population (75%) were more than 60 years old. Although the studies mentioned above are not directly comparable to the current study, they indicate the complexity of the adjustment process, which may progress as small steps over several years.

### Patients' most significant challenges at 12 months postoperative

The OAS subscore for sexuality (mean score of 3.20 12 months postoperative) indicated that this was the most challenging area for patients in our study. Sexuality is a multidimensional theme, including physical factors such as diagnosis, treatment and health [50] and psychosocial factors such as changes in body image and psychological, social and emotional aspects [51, 52]. Lifesaving cancer treatment such as surgery and eventual radiation therapy may have side effects such as nerve damage, resulting in erectile dysfunction or dyspareunia from reduced sensibility or anatomical changes [50]. In the current study, 59.4% of patients had cancer diagnoses. The nature of the study population could, therefore, be one explanation for low scores for sexuality. A Swedish study [4] also found low scores on the three OAS items about sexuality (item mean scores 2.1–3.9). Although the two studies are not comparable owing to different designs, most participants had cancer diagnoses in both, and low scores for sexuality were still demonstrated 12 months after the ostomy operation. Another explanation could be that patients and partners must adapt psychologically to bodily change, as shown in one review study [51]. For example, Vural et al. [52] studied the impact of ostomy on the sexual life of patients up to 5 years after surgery, and sexuality was still reported as a challenge. A longitudinal study among colorectal cancer patients found that patients with rectal cancer had marginally worse sexual function than those with other diagnoses, and it did not improve during the first six postoperative months. Body image distress was common, but this decreased significantly from baseline to 6 months [53]. This could explain the trivial improvements seen between 3 and 12 months postoperatively because adaptation processes are complex and may last several years. Several studies in a review study suggested a need for more counselling and education about sexuality [51], and another study indicates that SCNs need to know how patients wish to discuss sexuality [4]. Raising the topic of sexuality in consultations may be difficult for patients and SCNs. Thus, having a communication tool with which the patient can respond to concrete items about sexuality may be helpful.

Our study showed significant improvement in SF-36 scores for both sum scores (MCS and PCS) and the subscales of physical function, physical role function, pain, and emotional role function from 3 to 12 months postoperative. A previous Norwegian study also found lower SF-36 scores in the study population than in the general population, but effect sizes were small or trivial [8]. A Danish study found significantly better SF-36 scores six months postoperatively than at baseline in a patient group who attended a systematic education group than in those receiving standard follow-up. Those results were not compared to norms [15].

### Experiences from using the CFS in the ostomy outpatient clinic

Using questionnaires primarily made for research and not clinical may be somewhat challenging. In our study, patients responded to the OAS scale with single items and the answers were used in the subsequent consultation. We discussed the items with low scores first, and we had to improvise when items belonging to the same theme appeared several times and using single items in the clinic could be too complex. Thus, we divided the OAS into clinically meaningful subscales, including all the items in the scale [36] and used the subscales in our data analysis (Table 3).

A follow-up ostomy consultation has several components. Using the CFS was novel in that we had to seamlessly incorporate the answers shown on the screen during the consultation into the dialogue and simultaneously allow the patient to speak in their own words about everyday life with an ostomy. Using CFS in regular follow-up enables uncovering patient knowledge gaps and individual factors affecting their psychosocial health. The patient can respond to items on themes that may be difficult to raise otherwise during a consultation [54]. For example, the user panel's feedback indicated that using the questionnaires made it easier to discuss self-esteem/body image and psychological/existential factors, enabling them to be viewed as 'whole persons'.The patient and SCN can then communicate precisely to co-create new knowledge, gain insight, and share decisions [21]. Based on using PROs and clinical mapping, counselling and education may be more precise than without using such an instrument.

### Implications for practice and further use of CFS

The experiences from this study indicate that using CFS as a communication tool in the follow-up of ostomy patients is promising, as it may promote user involvement and prepare the SCN better for the consultation. Using single OAS items during the consultation was challenging, and we recommend that the tool is further developed using subscales instead of single items alone. Questionnaires that include subscales mirroring the patients' challenges, combined with recommendations and guidelines for follow-up and the SCNs' own experiences and knowledge, may enhance the follow-up consultation.

Another factor is the technology that can be enhanced, for example, by more accessible identification methods than BankID and by having items designed for response through mobile tablets. Accessing the questionnaires and answering them must be made as easy as possible so that patients can answer from home before their consultation. We also need to develop solutions for a better graphical presentation of the PROs during the consultation.

### Limitations and strengths

The current study had several limitations. First, the sample was limited, and the study was conducted in a single ostomy outpatient clinic. Second, the study lacked qualitative data about the patient's experiences and satisfaction with the PRO/CFS. Such data may have provided a more detailed view of CFS use in a clinical context. Third, we cannot claim that outcomes are better using CFS, owing to the study's observational design. The researcher (KLI) met some patients in the clinical follow-up consultation, which could be both a limitation and a strength. The limitation was that it could influence the patient's answers, especially on the GS-PEQ. A limitation was also that 29 of the 69 participants responded to a non-validated scale and those responses were not analysed.

A strength was the close collaboration between the developers of the CFS system and the clinic. Another strength was the long-term, continuous development of the CFS system in cooperation between patients, SCNs, researchers, and developers. Another strength was the general high response rate, except of the subscale “sexuality”, having a response rate of 64%.

## Conclusion

Our initial experiences and findings from using the CFS are promising, with SCNs suggesting that the CFS may lead to a greater in-depth discussion of patient challenges. Better technological solutions are required to enhance the CFS, such as finding other user-friendly but secure identification methods than BankID, improving the design for smartphone and tablet responses, and developing better summarised reports for documentation in the electronic patient journal. Further studies are needed to evaluate future versions of the CFS for this patient group.

## Data Availability

The datasets used and/or analysed during the current study are available from the corresponding author on reasonable request.

## Abbreviations

CFS: Clinical feedback system
GS-PEQ: Generic short patient experiences questionnaire
HRQoL: Health-related quality of life
IBD: Inflammatory bowel disease
IC: Ileal conduit
OAS: Ostomy adjustment scale
PRO: Patient-reported outcomes
SCN: Stoma care nurse
SF-36: Short form 36
